# Macroglossia Caused by Venous Congestion in a Patient With Amyotrophic Lateral Sclerosis

**DOI:** 10.1002/ccr3.71122

**Published:** 2025-10-07

**Authors:** Shunsuke Hino, Yosuke Iijima, Nami Nakayama, Norio Horie, Takahiro Kaneko

**Affiliations:** ^1^ Department of Oral and Maxillofacial Surgery, Saitama Medical Center Saitama Medical University Saitama Japan

**Keywords:** amyotrophic lateral sclerosis (ALS), general anesthesia, macroglossia, venous congestion

## Abstract

The macroglossia in this patient appeared largely related to impaired venous return from the tongue and neck after coil embolisation, rather than to tongue pseudohypertrophy due to denervation atrophy with fatty replacement caused by ALS.

## 1 Introduction

Macroglossia is defined as unusual enlargement of the tongue, and the etiology is diverse and includes a variety of congenital and acquired diseases [1]. Macroglossia is rarely encountered in oral surgery clinical practice. Amyotrophic lateral sclerosis (ALS) is a disease of unknown cause that occurs mainly in middle age and later in life. This rapidly progressive and fatal neurodegenerative disorder of the motor neurons is characterized by focal onset of muscle weakness and inexorable disease progression [2]. The development of macroglossia is reportedly not uncommon in ALS patients [3].

We describe herein the case of a patient on a ventilator with ALS, who did not previously have macroglossia but developed this pathology after coil embolisation for subarachnoid hemorrhage (SAH) due to the rupture of an anterior communicating artery aneurysm (Acom An).

## 2 Case Report

The patient was a 59‐year‐old woman who had developed ALS 9 years earlier. She had undergone gastrostomy 7 years earlier and placement of a tracheostomy 5 years earlier, and continued to receive treatment at home. Other medical history included syringomyelia and dyslipidaemia. No history of tongue swelling or macroglossia was evident. Three days before this presentation, she had developed a headache and subsequently became unable to communicate (progression from stage III), only communicates using non‐verbal yes/no responses, to stage V, unable to communicate by any means [4] and was taken to a local hospital where she was diagnosed with SAH due to rupture of Acom An based on computed tomography findings. The same day, the patient was transferred to the emergency department of this hospital for treatment of SAH. The next day (hospital day 2), coil embolisation was performed in the emergency department for SAH due to ruptured Acom An. On hospital day 4, she was referred to the Department of Oral Health Care Management due to poor oral hygiene and for prevention of aspiration pneumonia.

On initial examination in the department, the patient had a strong bite and extreme difficulty was encountered opening her mouth. No tongue swelling or protrusion of the tongue from the mouth was evident. Remaining teeth, including C4, were present in the anterior maxilla and the whole mandible. Oral function management was subsequently incorporated into the patient schedule.

## 3 Investigations and Treatment

**FIGURE 1:**
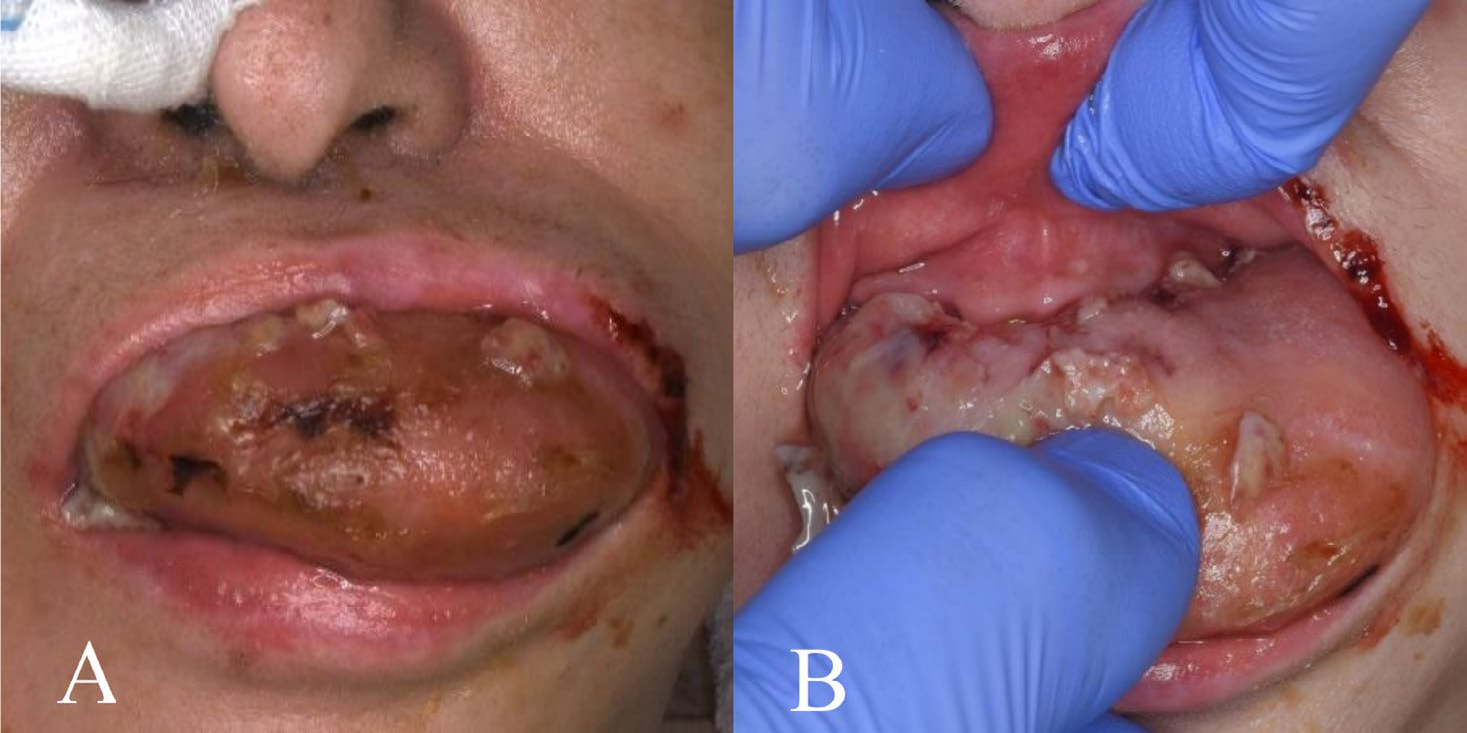
Facial and intraoral photographs on day 9. (A) Macroglossia is evident and the tongue protrudes beyond the oral cavity. (B) Bite wounds caused by the teeth are observed on the dorsal surface of the tongue.

**FIGURE 2:**
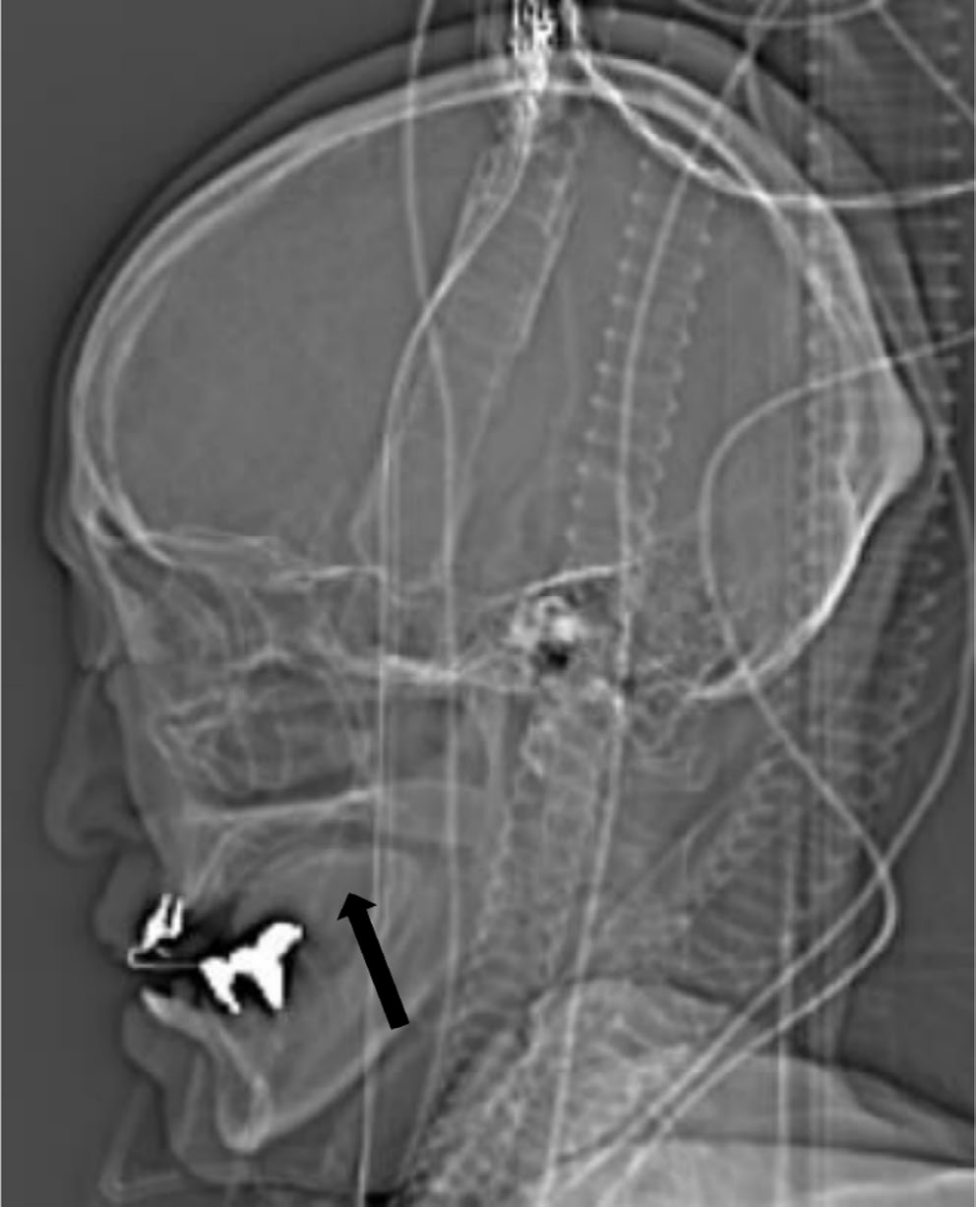
Computed tomography image taken after the onset of subarachnoid hemorrhage at the referring hospital. The tongue (arrow) is stored in the oral cavity.

**FIGURE 3:**
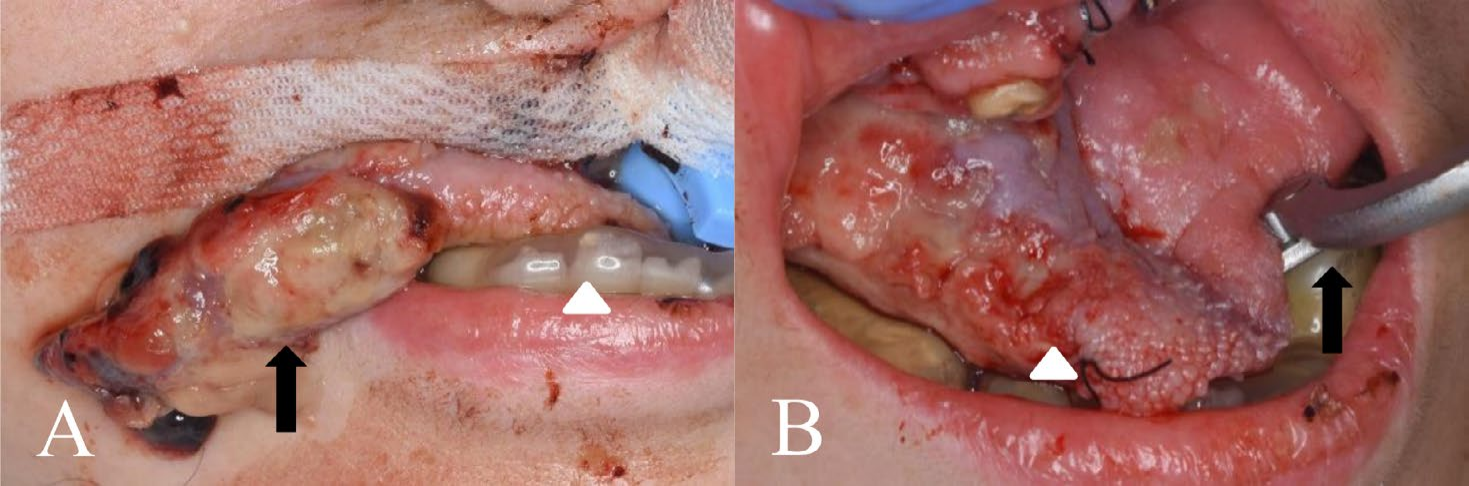
Facial and intraoral photographs on day 17. (A) The right lateral border of the tongue is torn due to biting (arrow). Mandibular splint (open arrowhead). (B) Intraoral view after removal of the torn fragment (open arrowhead). Bite block (arrow).

On hospital day 9, a visit to the ward for oral function management revealed a swollen, partially protruding tongue with severe difficulty opening the mouth (Figure 1). Upon reviewing the computed tomography images taken after the onset of SAH sent from the referring hospital, it was confirmed that the tongue was located within the oral cavity. Furthermore, since there was no swelling of the tongue prior to surgery at our hospital, it was confirmed that the swelling of the tongue occurred after surgery (Figure 2). Bite wounds caused by the teeth on the dorsal and inferior surfaces of the tongue were present, but no obvious persistent hemorrhage was observed. Massaging the tongue resulted in a slight but definite reduction in swelling. No other abnormalities were found in the oral region. On hospital day 10, the upper left central and lateral incisors were extracted and the sharp edges of the upper right canine, and right central and lateral incisors were abraded. The mandibular dentition was fitted with a splint, as bite injuries to the inferior surface of the tongue were more severe. Swelling of the tongue then reduced markedly. However, on hospital day 17, the right lateral margin of the tongue was torn (10 × 35 mm) and was excised (Figure 3). This was thought to be due to the removal of the bite block before the tongue was fully settled inside the dentition. The bite block was reattached and the patient was repositioned.

## 4 Outcome and Follow‐Up

On hospital day 18, the tongue showed a considerable reduction in volume and the likelihood of misbite was considered very low. The bite block was removed and the patient was observed. On hospital day 20, the tongue was seen to be settled in the oral cavity with no misbite. The patient was transferred back to the local hospital from which she had been referred.

## 5 Discussion

In this report, we described the case of a patient with ALS on ventilation who developed macroglossia due to impaired blood flow to the tongue after coil embolisation for SAH caused by Acom An rupture.

Macroglossia has been reported in patients with advanced‐stage ALS [5]. Matsuda et al. reported that macroglossia was present in 33.8% of patients with ALS, significantly correlated with higher body mass index, communication impairment, and long‐term use of tracheostomy‐invasive ventilation [3]. McKee et al. explained the mechanism underlying the development of macroglossia in ALS as pseudohypertrophy of the tongue due to denervation atrophy with fat replacement and obstruction of the veins and/or lymphatic vessels due to compression [5].

In exploring the mechanisms underlying macroglossia in the present case, the involvement of tongue pseudohypertrophy due to denervation atrophy with fat replacement was thus not possible to determine. However, the absence of macroglossia before coil embolisation, the improvement of swelling with massage of the tongue, and the absence of signs of allergy or acute inflammation causing the swelling led us to conclude that the macroglossia was instead due to venous congestion [6]. Venous congestion of the tongue and neck has previously been reported after general anesthesia [7, 8].

More specifically, the trigger for macroglossia in this case appears to have been impaired reflux due to compression of the veins in the tongue and neck, caused by the intubation tube and other intraoral inserts and body position in the perioperative period of coil embolisation. Subsequently, the swelling of the tongue made the tongue itself more vulnerable to biting, resulting in further strong compression on the veins inside the tongue (particularly the sublingual and deep lingual veins in the present case), further reducing venous return and probably exacerbating the tongue congestion. In cases of impaired venous return, macroglossia may appear immediately or, as in the present case, after several days [7, 9]. Of course, lymphatic insufficiency is also possible, but since the swelling is relatively rapid and substantial, venous insufficiency was considered the more likely cause of macroglossia in the present case [10].

The first way to improve macroglossia caused by impaired blood flow is to shift the position of any sources of compression that are present, such as intubation tubes, or to change the body posture if the patient is constantly in the same position. The tongue itself can be protected from compression by the teeth by using an aperture or bite block to maintain the opening of the mouth. However, positioning of these instruments may also impede venous return, so occasional repositioning is needed. Temporary reductions in the volume of the tongue can also be achieved by increasing blood return through massage. If residual teeth are present and clear bite marks are evident on the tongue, splinting may be effective. The form of the splint may need to take into account the elevation of occlusion to increase the volume of the oral cavity. Once treatment is initiated, the effects may become immediately apparent [6]. However, complete resolution of the macroglossia can take several days, with some reports describing periods of up to 3 weeks before complete normalization [9]. In this case, the right lateral margin of the tongue was torn as a result of incorrectly timed treatment and premature removal of the bite block. Table 1 summarizes the clinical course, etiology, treatment, and prognosis of ALS patients with macroglossia.

**TABLE 1:** Summarizes of ALS patients with macroglossia.

Author/year	Patient background	Onset timing	Suspected etiology	Treatment	Outcome
Matsuda et al., 2016 [3]	ALS, advanced stage, tracheostomy‐invasive ventilation	Gradual (months–years)	Pseudohypertrophy due to denervation atrophy with fatty replacement; possible chronic venous/lymphatic stasis	Supportive oral care, prevention of trauma	Persistent tongue enlargement
McKee et al., 2013 [5]	ALS, late stage	Chronic	Pseudohypertrophy from denervation; possible venous/lymphatic obstruction	Supportive care	No significant size reduction
Miura et al., 1996 [8]	Non‐ALS, spinal surgery	Immediate postoperative	Venous congestion from prone positioning, airway compression	Position change, airway adjustment	Resolution within 48 h
Present case (Hino et al., 2025)	ALS, ventilator‐dependent, post‐coil embolisation for SAH	Delayed onset (day 9)	Venous congestion from perioperative tube/positioning, aggravated by tongue biting; pseudohypertrophy excluded based on rapid reversibility	Bite block, splint, tongue massage, tooth extraction/abrasion	Resolution within ~11 days

In conclusion, this report describes the case of a patient with ALS on a ventilator who developed macroglossia after coil embolisation for SAH due to a ruptured Acom An. The macroglossia in this patient was suggested to be largely related to impaired venous return from the tongue and neck after coil embolisation, rather than tongue pseudohypertrophy due to denervation atrophy with fatty replacement caused by ALS.

## Author Contributions

**Shunsuke Hino:** conceptualization, writing – original draft. **Yosuke Iijima:** data curation, writing – original draft. **Nami Nakayama:** data curation, supervision, writing – review and editing. **Norio Horie:** conceptualization, project administration, writing – review and editing. **Takahiro Kaneko:** conceptualization, project administration, supervision, writing – review and editing.

## Consent

Written informed consent was obtained from the patient to publish this report in accordance with the journal's patient consent policy.

## Conflicts of Interest

The authors declare no conflicts of interest.

## Data Availability

The data that support the findings of this study are available from the corresponding author upon reasonable request.
